# Combining elements of the CO-OP Approach^™^ with education to promote healthy eating among older adults: A pilot study

**DOI:** 10.3389/fresc.2022.971300

**Published:** 2022-10-21

**Authors:** Deirdre R. Dawson, Yael Bar, Fatim Ajwani, Shlomit Rotenberg, Barbara Atlas, Maria Ricupero, Carol Greewood, Matthew D. Parrott

**Affiliations:** ^1^ Rotman Research Institute, Baycrest Health Sciences, Toronto, ON, Canada; ^2^ Department of Occupational Science / Occupational Therapy, University of Toronto, Toronto, ON, Canada; ^3^ Rehabilitation Sciences Institute, University of Toronto, Toronto, ON, Canada; ^4^ Cardiovascular Prevention and Rehabilitation Program, Toronto Rehabilitation Institute, University Health Network, Toronto, ON, Canada; ^5^ Dalla Lana School of Public Health, University of Toronto, Toronto, ON, Canada; ^6^ Department of Nutritional Sciences, University of Toronto, Toronto, ON, Canada; ^7^ PERFORM Centre, Concordia University, Montreal, QC, Canada

**Keywords:** diet intervention, CO-OP Approach™, aging, pilot study, brain health

## Abstract

This paper describes an exploratory study developing the Baycrest Brain-healthy Eating Approach (BBEA). Poor diet is a modifiable risk factor for many health problems including dementia. Mediterranean type diets, high in plant-based foods, rich in poly- and mono- unsaturated fatty acids with minimal consumption of saturated fat, red meat, and processed foods, are considered brain healthful. While several dementia prevention trials randomized controlled trials have included nutritional counselling in favor of these diets as one component of their interventions, the extent to which dietary change occurred is not known. Based on observations that a strategy training approach, the Cognitive Orientation to daily Occupational Performance (CO-OP) Approach, was beneficial for promoting lifestyle changes in older adults with complaints of cognitive changes, we undertook to develop the BBEA combining elements of CO-OP with didactic nutrition education. This exploratory, descriptive study assesses the feasibility and acceptability of the BBEA. Healthy community dwelling older adults (*n* = 5) were recruited using convenience sampling. Participants received five, 2 h, group sessions. During these sessions participants were supported in adopting dietary practices consistent with brain healthy eating. Each participant set specific dietary goals important to them. Feasibility of the intervention was demonstrated through high levels of attendance and by the findings that at each session, all participants set personally meaningful goals and received education on selected brain healthy eating topics. Acceptability was demonstrated through participants' positive reports of their experiences and perspectives obtained *via* semi-structured interviews. Thus, the BBEA appears to be feasible and acceptable.

## Introduction

The World Health Organization estimates there are 55 million people worldwide that live with dementia and nearly 10 million new cases annually (1). Given the enormity of this public health problem, delaying the onset and/or preventing dementia entirely is the focus of many researchers. To date, cures for dementia have not been found, however, a variety of risk factors that are modifiable have been identified including social and physical inactivity, obesity and diabetes (2). The importance of implementing strategies to improve uptake of behaviors that will modify such risk factors is immense. The Alzheimer's Association has estimated that a treatment implemented in 2025 that delayed the onset of Alzheimer's disease, the most prevalent form of dementia, by 5 years, would by 2030 save the United States over 83 billion dollars in the US (3). The savings in personal and familial distress, while incalculable, would also be great.

This paper focuses on one modifiable risk factor: poor diet. It is widely known that a poor diet contributes to obesity and diabetes. However, diet has also been implicated in cognitive impairment and Alzheimer's disease in the absence of obesity and diabetes. Accumulating evidence shows that Mediterranean type and low glycemic index diets are associated with reduced Amyloid-beta protein burden (an Alzheimer disease biomarker) in cognitively normal older adults and those with mild cognitive impairment (4–6). These diets, considered brain healthful diets, are high in plant-based foods, rich in poly- and mono- unsaturated fatty acids and minimize consumption of saturated fat, red meat, and processed foods (7). Adoption of brain-healthful diets has been investigated as a way of delaying and/or preventing cognitive decline and dementia in seniors.

While some of the recent trials on diet and cognitive change have shown benefit, the reported benefits are typically small (8). The Nutrition for Dementia Prevention Working Group recently explored potential reasons for these small effects and suggested lack of adherence to the intervention as one potential reason (8). Adherence in relation to diet and nutrition, in the two largest multidomain dementia prevention intervention studies, was measured as attendance at sessions and pill count for the study using supplements (9). Thus, whether people made dietary changes is unknown. For the more successful trial, the Finnish Geriatric Intervention Study to Prevent Cognitive Impairment and Disability (FINGER), the nutritional intervention combined education and counselling (10). Many other trials investigating the effects of diet on cognition have also relied on education and/or counselling to try to promote changes in eating patterns (e.g., 11–16). As behavior change is notoriously difficult, we questioned whether other approaches to promoting diet change might be developed that would be more effective.

Answering this question was particularly pertinent for us, as we were part of the Canadian Consortium on Neurodegeneration in Aging and focused on developing and testing novel approaches for dementia prevention. Some of our group had previously demonstrated that a strategy training approach, the Cognitive Orientation to daily Occupational Therapy (CO-OP) Approach^™^ was beneficial for promoting lifestyle changes in older adults with complaints of cognitive changes but no objective evidence of cognitive change (17). Based on this, we wondered whether we could combine strategy training with nutritional education and counselling would be feasible and ultimately whether it would help promote positive diet change and prevent dementia.

Thus, the objectives of this exploratory study were to develop and investigate the feasibility and acceptability of a novel intervention that we hoped would ultimately be used in trials promoting adoption of eating patterns known to be brain healthy. As this study was exploratory and descriptive, we did not have specific hypotheses.

## Methods

### Design

For this exploratory study, we used a qualitative, descriptive design to explore participants' experiences and perspectives of their participation. This design fosters a low-level of interpretation of the data unlike other forms of qualitative research in which the researcher may choose a conceptual or philosophical framework (18). This type of design allows the researcher to provide a comprehensive summary of events and is particularly useful for asking who, what and where questions (18).

Ethics approval was obtained from the Baycrest Research Ethics Board (REB), all participants provided informed, written consent, and all procedures and analysis were conducted according to the ethics standards of the Baycrest REB and with the 1964 Helsinki Declaration.

### Participants

The study used a convenience sample. Participants were community-dwelling, older adults who were volunteers at Baycrest Health Sciences, Toronto, Canada. The Director of Volunteer Services informed volunteers about the study. Interested individuals met with a trained research assistant who provided written information about the study and invited consent. Inclusion criteria were that participants self-identify as healthy, be fluent in spoken and written English and be available to participate in the intervention.

### Procedure and timeline

Following recruitment, participants met for 5 weeks with 1-, 2-h session per week. The sessions were facilitated by registered dietitians (FA, MR) and trained CO-OP Approach^™^ therapists (DD, YB). Session content is described in detail below (see Intervention). Individual nutrition counselling sessions of 20–30 min with a dietician (FA, MR) were available in weeks 3 or 4. Attendance was recorded at each session.

Following completion of the intervention, semi-structured interviews were conducted by a trained interviewer (BA) who was not involved in facilitating the intervention (see Measures for description of interview content). Interviews lasted 60–90 min and were audio-recorded and transcribed verbatim using InqScribe® software (19).

### Measures

Basic sociodemographic data (age, education, marital status) were collected from each participant. While cognitive status was not formally measured, all participants were functioning independently including participating in volunteer work at Baycrest Health Sciences.

Feasibility was measured by attendance and completion of the intervention. Acceptability was examined qualitatively through eliciting participants' experiences and perspectives the post-intervention interviews. The interview guide provided some structure while allowing participants the flexibility to offer perspectives that might otherwise not be considered by the researcher. Question probes included asking participants what their understanding of the purpose of the group session was, what was important for them in the sessions, what dietary changes they made, what dietary changes they made, which aspects of the intervention that served as facilitators or barriers to dietary change; and suggestions for adaptations that would lead to sustainability of dietary change. They were also asked for their perspectives on the structure and facilitation of intervention and their involvement in it.

#### Intervention

The Baycrest Brain Healthy Eating Approach (BBEA) was developed by combining the Brain Health Food Guide (20) with elements of the CO-OP Approach™. The Brain Health Food Guide is an evidence-based approach to healthy eating based on a Mediterranean diet. The CO-OP Approach is one in which participants work on achieving self-identified goals by applying a meta-cognitive problem-solving strategy (GOAL-PLAN-DO-CHECK). Facilitators guide participants to develop personally and contextually relevant plans and goal specific strategies as they work towards goal attainment (21). Previously, we conducted a small, randomized controlled trial (*n* = 19) to determine if use of this approach would result in meaningful everyday life changes in healthy older adults who were objectively cognitively intact but had some cognitive complaints (e.g., starting to do one thing at home and unintentionally getting distracted into doing something else). We found that those in the experimental group made clinically significant improvements on more than 60% of their self-identified goals (e.g., to exercise five times/week) relative to only 33% in the control group (17). These promising results led us to consider combining elements of this approach with brain-healthy eating.

The resultant BBEA includes nine components listed and described in Table 1. The first element, group support through a small group setting, was included based on investigators' clinical and research experience and the known benefits of group support (24, 25). The BBEA includes three brain-healthy eating components the food guide (20), education, and individual nutrition counselling. The four components selected from the CO-OP Approach have previously been posited as essential elements for its efficacy (26). Having participants actively involved in setting their own dietary goals provided maximal salience and a context for behavior change that is meaningful and relevant. While goal setting is often associated with making dietary change (22), it is not standardly included in all dietary studies and, to our knowledge it has not been combined with the other CO-OP elements described here.

**Table 1:** Components of the Baycrest Brain-Health Eating Approach*.

Components	Description
1. Group support	The intervention is developed in a small group setting that encourages group support. Each 2-h session includes goal setting, didactic education and a brain-healthy snack (fruit and water) during the break.
**Brain-healthy eating elements**	
2. Didactic education	Education focused on the health benefits of particular foods, suggestions for how these foods might be incorporated into one's diet and making healthy food choices. Topics were selected collaboratively by participants.
3. Individual nutritional counseling	An individual 30-min session with the dietitian was offered to address individual concerns and barriers to change.
4. Brain health food guide (BHFG)	The BHFG, developed in 2016, is designed to be a practical life-long eating guide and is based on evidence from European and American epidemiologic and clinical trial results related to diet and cognitive function or dementia risk.
**CO-OP Approach™ elements**	
5. Individualized goal setting	Participants set weekly goals developed to align their diets more closely with the BHFG (e.g., Eat berries 3x/week).
6. Guided discovery	Rather than group leaders (GL) telling participants what goals to set and how to achieve them, they used a series of questions to allow participants to identify individualized plans that would foster their goal achievement (e.g., GL: What will help ensure you eat 3 servings of berries each week?)
7. Cognitive strategy use	A meta-cognitive strategy was used to support goal-setting and behavior change (GOAL-PLAD-DO-CHECK). Other cognitive strategies were used to support specific goals (e.g., post-it note on fridge reminding participant to use blueberries in smoothies).
8. Dynamic performance analysis	The GL guides/fosters discussion about plans to encourage participants to consider their feasibility making setting plans an iterative process and modeling this iterative process to encourage participants to continue it on their own.
**Written materials**	
9. Participant binders	Each participant was provided with a binder containing the BHFG, tracking sheets (22) to record their goals, plans and progress and copies of power-points related to the didactic education.

*Adapted from Table 1 in Koblinsky and colleagues (23).

The process of using CO-OP in this group setting was as follows. After individual goals were set, participants were involved in an iterative process of defining concrete plans to achieve their goals, and self-monitor their progress. We consider that the participants' work of “discovering” successful plans also may be integral to successful behavioral change and contributes to participants' self-efficacy as they attribute their successes to their own work. While the group leader occasionally offered suggestions based on their knowledge and experience, participants decided whether they would use these suggestions and how they would implement them in their daily lives. For example, in relation to a goal of eating berries three times per week, a participant initially planned to do so by putting them in breakfast smoothies, then later developed a new plan to include them in their cereal when they didn't want smoothies.

Each of the five weekly sessions started with participants reviewing progress on goals set the previous week, discussing of whether the goal was partially, or fully met and whether the plan made to achieve the goal “worked” or whether a new plan was needed. If changes or new plans were needed, group leaders used guided discovery methods to facilitate participants development of individualized plans that could be readily incorporated into their daily routine and refrained from than telling them what to do. Following a break with a brain-healthy snack provided (fruit, water), a group leader provided didactic education on topics related to brain-healthy eating. In the final part of each session, each participant set one or more new goals with plans, related to further adoption of brain-healthy eating approaches. All sessions with individual nutritional counselling provided by a dietitian.

The intervention was supported by written materials provided to participants. These were in a standard format for each participant but were the participants' property and could be adapted by each person as they wished. For this pilot study, participants were offered a range of topics for the didactic education and collaboratively selected four: incorporating legumes into one's diet, health benefits of various plant foods, healthy fats, and label reading.

### Analysis

Attendance was taken at each session. Group leaders' notes were analyzed to determine if the weekly content proceeded as per the planned session design. Interviews were analyzed using an essentialist approach meaning participants' own words were used to generate codes, sub-themes and overall themes through an iterative approach of assigning and reviewing initial codes, generating, reviewing and defining themes and then connecting themes to provide an account for the data (27). To ensure rigor, two authors (FA, YB) assigned codes and generated themes independently and then reviewed them and held regular discussions with other team members (DD, CG) to finalize themes.

## Results

Five women (mean age: 71.5 ± 6.2, mean years education 17 ± 3.7) participated in the study and follow up interviews (P1, P2, P3, P4, P5). Three lived alone, two with their spouse. All reported doing their own cooking and grocery shopping. Feasibility of the intervention was demonstrated through high levels of attendance: Four of the five participants attended all group sessions; one participant declined the individual counselling. Group leaders' notes confirmed the feasibility of the session format design as all components were able to be included in the 2-h sessions, all participants had the opportunity to set goals each week and education on brain healthy eating was included at each session with the exception of the introductory session.

Acceptability was demonstrated through the content of the interviews with the participants. The thematic analysis from these interviews revealed four overarching themes. Three themes were understood as contributing to the intervention being effective. The fourth, *Elements for Sustainability*, related to the intervention being effective in the longer term. Table 2 includes theme and sub-theme descriptions and quotes which depict and elaborate on these.

**Table 2:** Themes, subthemes and supporting quotes*.

Main themes and description	Subthemes and quotes
**Perceived Active Ingredients** (elements of the intervention participants found helpful.)	**Personal desire for change** *I had dementia roots on my dad's side of the family … and I was experiencing myself becoming somewhat more forgetful, and so I wanted to get a sense of how I could keep myself mentally alert and look at diet. Look at the foods I eat as a way of maintaining my sense of alertness* (P3) **Credibility and professional support** *… Particularly the dietitians who came with specific information that you could take home which was immediately useful and … I found that piece that was very very good … and the fact that there were people that give informed information* (P2) *Yes, I took some of it in and I thought they were very good, and they were not judgemental which I felt … not that I thought they would be but you know maybe it's coming from within my head “Oh you know, maybe they're judging me.” But they weren't, for sure. But uh you know every week I thought they were excellent, and I took in you know everything they said and yeah it was very helpful* (P4) **CO-OP Approach Elements** *… it was really nice to see it at each session, each one setting a goal and coming back the e next week and seeing if they followed it and most of us followed that pretty well. So, it was good, yeah* (P5) *… you can have a plan, or a goal, but how do you get to that goal? So, you do have to have a plan and you know what am I going to do today, what am I going to do, how am I going to get there … you know there are different steps to get there, and yes … I found that good* (P5) *I think an important part of the group can be or should be is that one supports the individual choices [i.e., goals] that people make in their diet and in their eating* (P2)
**Suggested Active Ingredients** (elements that participants felt may have increased its benefits.)	**Individualizing the approach** *… and maybe even asking people what their food issues are, and I mean I don't know if people in this group were bulimic or had … ever had an eating disorder. But even knowing a little bit of people's prior history before the [group] for the people who were leading the group could have been helpful* (P3) *… I think that taking in mind how adults how seniors learn. Okay and looking at the different sort of the background about when you learn things how do you like to learn them. There's a whole bunch of learning style inventories that are very simple* (P3) **Expanding the availability of support** *… more peer support would be helpful, and I think more individual support, but from another group member. So, you can just call someone and say I'm really stuck, I don't know what to do, you know whatever. Could talk with a buddy about food* (P4)
**Barriers to Address** (elements of the intervention participants identified as needing change.)	**Intervention burden** *… [completing the] food sheet was very good the first week … I guess after a while you kind of get to know what you're supposed to eat, what you're not, how much you're supposed to eat so it's [the food tracker] not so important as at the beginning* (P5) **Group management** *… and I wish we would have started the lecture first, and then we seemed to be rushed at the end to set our new goals* (P1)
**Elements for Sustainability** (elements of the intervention participants identified as important for sustaining dietary change.)	**Awareness about brain healthy eating** *… it just clarified a lot of stuff … yeah, I thought what they brought in was very useful … and I think I learned some new things that I didn't know* (P1) **Doable and practical diet approach** *…, it's [the BHFG] something I think you could stick with because it's not really life-changing … I think it's something that you could live with more* (P1) *And we were given guides of how much and when to eat things. But it was pretty good, and it was also more flexible because with a lot of things that I have personally been on, you're restricted from a lot of things … you're always tempted and you always want these things whereas eating while here … learning to eat this way, sure you're allowed to have a little bit of this and a little bit of that and long as you don't go overboard* (P5) **Self-directed approach to goal achievement** *Every week they had set a goal, and I think that was good because then I tried to work towards one thing at a time. Well, you know the first week was to eat more fruit. And then I tried to keep it the second week but then I added something else new. So, I wasn't going “gun-ho” right away. I think that's you know unrealistic* (P1) *Um yes, I mean you have … your own plan, I mean no one can tell you how to, I mean I guess you know they can suggest some things, but you know it has to be what is comfortable for you and what is convenient for your own lifestyle* (P2)

*For clarity, repeated words and interjections (e.g., um, uh) have been removed and words in square brackets have been added.

All participants commented on helpful aspects of sessions leading to theme 1, ***Perceived Active Ingredients***. They described their *personal desire for change* as being important and arising specifically from a desire to reduce their dementia risk. They valued having Registered Dietitians provide the education and professional support received in the groups and they valued the goal-setting focus. They also spoke about the importance of setting personally meaningful dietary goals stating.

Theme 2, ***Suggested Active Ingredients***, includes participants' views of elements they believed would enhance intervention efficacy including further opportunities for *individualizing the approach*, providing facilitators with more information about individuals prior to the group start (as they believed this contextualization would have helped the group process), and *expanding the availability of supports* to complement support provided by the group, for example, adding a peer support mechanism.

The third theme, ***Barriers to Address***, encompassed factors participants perceived as hindering efficacy. Participants expressed that tracking their eating habits was initially helpful, but by the second or third week was experienced as *burdensome*. They also proposed better *group session management* particularly as it related to time to ensure equitable distribution between participants to allow all participants to discuss their individual goals.

Theme 4, ***Elements for Sustainability*** captures participants' reflections on how the immediate benefits of the intervention might be fostered to result in more lasting change. Participants identified three elements they believed to be critical: developing *knowledge and awareness of brain-healthy eating*, that *the BBEA was flexible and doable* as it was similar to a Mediterranean diet, and that it was *self-directed*, that is they could determine how they would change their own diets to be more aligned with the Brain Health Food Guide.

**Figure 1:**
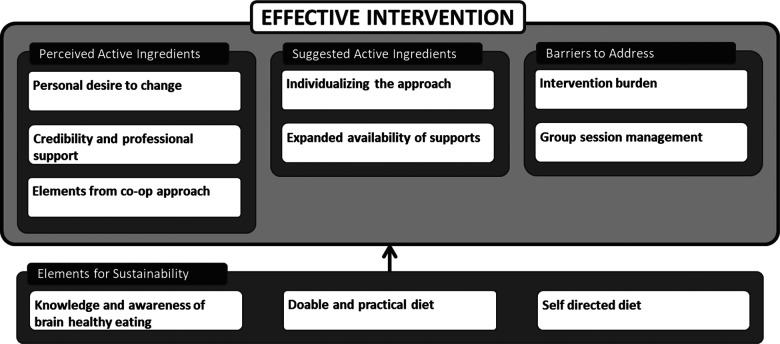
Posited ingredients and elements necessary for an effective and sustainable brain healthy eating intervention.

Based on these themes, we developed a model positing the ingredients necessary for the intervention to be effective and sustainable (see Figure 1).

## Discussion

This paper describes the development of the Baycrest Brain-healthy Eating Approach (BBEA) and provides findings on feasibility and acceptability. We found the intervention feasible based on high attendance and group leaders' ability to administer the content and discussions in the planned timeframe; and acceptable based on participants' largely positive experiences.

Participants' perspectives about the intervention were represented by four themes: *perceived active ingredients*, *suggested active ingredients*, *barriers to address* and *elements for sustainability* from which we developed a thematic map around the hypothesis of the BBEA being effective (see Figure 1). The map portrays participants' views that the intervention was short (5 sessions) but beneficial. Comments about sustainability should be understood as suppositions and the entire map as a hypothesized framework for the potential efficacy of the BBEA.

As this was the first-time elements of the CO-OP Approach were incorporated into a dietary intervention, we were encouraged that participants found them to be important in supporting their adoption of the BBEA (Components 5–8, Table 1). Participants referred to positive experiences with the facilitators' use of guided discovery which they perceived as non-judgmental, their use of the cognitive strategies and the goal setting element. Participants were positive about the diet being doable and flexible, that it was self-directed, the group support and the responsiveness of the facilitators. These themes are similar to those reported elsewhere in studies investigating participants views regarding dietary changes. For example, social support in the form of group and/or peer support has been reported as valued in studies focusing on promoting adherence to Mediterranean-type diets (28, 29). More peer support was desired by our participants. We anticipate that over the course of a longer intervention this might develop naturally but could also be facilitated.

Improved knowledge and awareness of brain-healthy eating, flexibility and self-directedness of the approach were seen as supporting the sustainability of behavioral change over time. This is in line with Self Determination Theory, that maintains that people are intrinsically motivated through a need for autonomy and choice and mastery (30). Self-determination has previously been achieved in health interventions for older adults by engaging clients in decisions that affect the intervention (31), and through enhancing their understanding of clinical reasoning and the rationale behind suggested behavioural changes (32). We believe that using guided discovery to facilitate participants setting their own goals and developing their own plans and asking them to select the topics they were most interested in for education, contributed to the self-directedness they express.

While we designed this intervention to be highly individualized, further “customization” was recommended by participants in relation to group leaders having more knowledge about their individual dietary situations and to their preferred learning style. We did not collect a medical or detailed dietary history and agree with the participants that this could be helpful information for group leaders. The pilot nature of the study precluded adapting written materials for each participant.

Subsequent to this exploratory study, the BBEA was included in a small, pilot randomized controlled trial (*n* = 14) investigating the feasibility and preliminary efficacy of exercise and the BBEA on hippocampal volume among older adults who were at risk for dementia (23). While no significant changes in hippocampal volume were seen, participants in the experimental arm did report substantial improvements in their diet. The results of this trial suggest the BBEA may have benefit for promoting dietary change.

### Limitations

This was a very small exploratory study with a homogenous and highly educated group of participants. Further, while all participants self-identified as healthy, it is possible that some may have met the criteria for subjective cognitive impairment or have had objective markers for cognitive impairment. These limitations mean that similar questions posed to a larger, more diverse group including those with cognitive impairment will likely reveal additional perspectives and experiences. In addition, as this study including only five sessions, it may be that interviewing individuals involved with a longer intervention would provide additional recommendations for intervention change and/or barriers to adherence.

## Conclusion

We undertook this pilot study to determine whether the BBEA was feasible and acceptable to older adults with the view of testing it further in randomized controlled trials (RCT). We established the feasibility and acceptability of the pilot version of the BBEA and participants experienced it positively. We believe it extends the literature on dietary intervention design by suggesting additional elements that may be helpful.

## Data Availability

The raw data supporting the conclusions of this article will be made available by the authors to interested researchers, without undue reservation.
